# A replication of Triplett’s ‘social facilitation experiment’

**DOI:** 10.1038/s41598-025-25608-x

**Published:** 2025-11-18

**Authors:** Cillian McHugh, Siobhán M. Griffin, Elaine L. Kinsella, Michael Quayle, Bob Strunz, Orla T. Muldoon

**Affiliations:** 1 Centre for Social Issues Research, Department of Psychology, University of Limerick, Limerick, Ireland; 2 Centre for Robotics and Intelligent Systems, Department of Electronics and Computer Engineering, University of Limerick, Limerick, Ireland; 3 School of Psychology, Queens University of Belfast, Belfast, UK

**Keywords:** Group influence, Performance, Replication, Social facilitation, Triplett, Human behaviour, Social evolution

## Abstract

**Abstract:**

A seminal study by Triplett provided initial evidence for the social facilitation effect, and over the last century, research has demonstrated this effect across a range of settings. However, despite the importance attributed to Triplett’s study, no research has replicated the original study paradigm. Furthermore, in the context of research advances and methodological rigour, some elements of the original study weaken the validity of the observed findings. We address these critical limitations and replicate the original study, employing a standardized study protocol to minimize potential confounds, such as practice effects. This research reports on 445 school-aged children (*M*_*age*_ = 10.8, *SD* = 1.3) who completed the study. As pre-registered in our Stage 1 submission, using a purpose-built apparatus, participants turned a crank to move a marker along a string course between two pulleys. Pairs of participants completed this task four times, twice alone and twice together. Trial order was standardized and balanced to control for possible order/practice effects. In line with Triplett’s original study, we show that participants completed the task faster in the together condition than in the alone condition. We also show interesting variability depending on age and gender, as well as demonstrating “carryover” effects. Thus, we replicate and extend this classic finding, adding to the literature on social facilitation/competitive coaction. In addition, the materials and methods developed here provide a template for future studies to further examine this phenomenon.

**Protocol registration:**

The Stage 1 protocol for this Registered Report is available at https://osf.io/vsgpq, and appears as accepted in principle by the journal on 10th August 2022.

## Introduction

In what is often referred to as the first experiment in social psychology^1^, Norman Triplett showed that completing a task in competition with another participant improved performance on the task compared to completing the task alone^2^. While Triplett presented this as an investigation of how competition can improve performance^2^, his seminal study has also been credited as giving rise to a rich literature on social facilitation, the phenomenon where the mere presence of others can lead to improvements in performance^3,4^. Indeed, at the time of writing (July, 2022) a search on PsychINFO for the term social facilitation results in over 3,244 hits and Triplett^2^ has been cited by over 2,122 published studies. Given the centrality of the original finding to the discipline, as well as the widespread value of the effect in sport and performance psychology^5^, it is surprising that the original study has not been replicated. Over a century later, the present research will conduct a replication of Triplett’s original experiment.

Triplett’s experiment stemmed from natural observations that cyclists performing in competition cycled faster than when alone. He theorized this could be due to several factors both physical (e.g., shelter from the wind) and psychological (e.g., encouragement, competition arousal). This led Triplett to design what is believed to be *one* of the first empirical social psychology experiments e.g.,^1,3^, though the claim that this is the “first” social psychology experiment is contested^6^. The focus of his study was on what Triplett termed “dynamogenic factors” - when the presence of another competitor stimulates competition arousal for a full review see^7^.

In Triplett’s study, children were asked to rotate a fishing reel to move a flag around a four-metre course; trials alternated between performing alone and performing in competition together with another child^2^. The results of this experiment demonstrated that, in general, children performed better when completing the competition trials together compared to performance alone (*n* = 40). However, there was some variation noted whereby ten children were described as “overstimulated,” performed slower on the together trials, and another ten children were not affected by the competition. Overall, his research suggested that competition served to stimulate performance on the task compared to completing the task alone^2^. Triplett’s original study has given rise to rich research traditions on both competition effects and audience effects on performance. Triplett’s study presents a clear demonstration of competitive coaction, whereby individuals work alongside one another, performing the same type of activity in competition with one another^8^. However, the impact of Triplett’s work extends beyond the demonstration of competition effects.

Two decades after Triplett’s study, Allport coined the term social facilitation to describe the phenomenon whereby performance on a task is positively influenced by the presence of others while completing the task^9^. Importantly, social facilitation can occur in different social presence contexts including having an observer or audience presence, having an evaluative audience or observer, a non-competing co-actor, or in the case of Triplett’s experimental study – a competing co-actor^10^.

Various theoretical explanations for social facilitation effects have been proposed, we will briefly discuss some main hypotheses, but for full reviews see;^4,11^. Building on Triplett’s work, Zajonc^12^ demonstrated that task complexity affects social facilitation, showing that social presence enhances performance on simple tasks, but hinders performance on more complex tasks — a finding that has since been shown in a variety of experimental settings amongst humans and other species. For example, during simple tasks, such as eating food, paired animals consume more compared to when eating alone e.g.,^13^. In the presence of other cockroaches, cockroaches navigate runways more quickly (simple task) and mazes more slowly (complex task), than when alone^14^. However, it is worth noting that an attempted replication of Zajonc^12^ did not fully replicate this effect; in both the simple and complex conditions the cockroaches performed more slowly when other cockroaches were present^15^.

Zajonc hypothesized that habits are energized by “generalized drive”, a position informed by behavior theory e.g.,^16,17^. In Zajonc’s view^12^, see also^4^, the presence of others leads to an increase in generalized drive thus facilitating habitualized dominant responses. A key strength of this explanation was its ability to accommodate the divergent results for simple versus complex tasks, whereby facilitation is observed for simple tasks but not for complex tasks. For simple tasks the dominant (habitual) response is the correct response, while for more complex tasks the dominant response may not be the correct response^4,12^. However, others have suggested that presence alone is not sufficient to lead to improved performance through increased drive^4,18,19^. Instead, Cottrell^18,19^ argues that it is the *expectation of evaluation* by others that increases drive, and that the apprehension associated with anticipating other’s evaluation that leads to the observed influences on behavior^4,18,20^.

Furthermore, some cognitive based approaches have emphasized the role of attention^21–24^. According to distraction-conflict theory^21,24^, when the social presence of others (co-actors or audience) is distracting, it can lead to attentional conflict, a form of response conflict regarding what attentional response one should make (paying attention to the focal task or to the person present), at least when the task is attention demanding. This conflict, in turn, may threaten the actor with cognitive overload and, ultimately, cause a restriction in attention focus. Ironically, attention focusing may produce just the task effects associated with the energization of dominant responses: (a) facilitation of performance (by screening out non-essential stimuli) when the task is simple or requires attention to a small number of central cues and (b) impairment of performance (by neglecting certain crucial stimuli) when the task is more complex or demands attention to a wide range of cues^21^. One strategy for differentiating the two hypotheses (attention focusing versus dominant response) is to use poorly learned tasks that involve only a few key stimuli. In this context, the attention-focusing hypothesis predicts social facilitation, whereas the dominant-response hypothesis predicts social impairment for such a strategy and results favoring Baron’s cognitive view of social presence effects see ^23^.

Moreover, Muller and Butera^25^ propose an approach that integrates distraction-conflict theory^21^ and social comparison theory^26^. According to this approach, people in coaction settings may experience self-evaluation threat which induces attentional focusing^25^. Specifically, when there is potential for a co-actor to be superior, this may pose a threat to a person’s self-evaluation, and this threat leads to attentional focusing (or increased drive).

Despite the theoretical debates that exist as to what drives the effects of social facilitation, research has highlighted its practical implications. Similar to animal research, humans have been found to consume more food in the presence of others, particularly in the presence of family and friends e.g.,^27^. The presence of others influences how people spend their money – people tend to donate more money while in the presence of others than when alone^28^, and buy more when shopping with others, than alone^29^. In terms of sport and competition, adults riding stationary bikes (enhanced with virtual reality equipment) exercised more when competitive avatars were introduced, if they scored high on self-reported competitiveness^30^. On the other hand, sometimes the presence of another can be detrimental. For instance, analysis of archival data shows that learner-drivers who completed their driving test (a complex task) with another individual awaiting their test were more likely to fail compared to learner-drivers who completed their test without a waiting observer^31^. Indeed, the effects of social facilitation are far-reaching and can extend to several domains, such as education (group work versus solo work, examinations to be taken alone or in examination halls), business (brainstorming, open-office planning), academia (writing retreats, writing groups) and fitness (e.g., group fitness classes).

### A critique of Triplett’s experiment: a case for replication

While Triplett’s classic study has been influential in inspiring later studies e.g.,^9,10,12^, shaping the direction of social psychology literature, and has featured in many psychology textbooks^32,33^, the original study has never been directly replicated. Furthermore, the original study can be critiqued in a number of ways. First, the sampling of participants was problematic. In the original study^2^ “the records of 225 persons of all ages” were taken (p. 520). Yet the findings presented by Triplett are based on the data of a subset of participants — 40 children (aged 8–17 years). It is unclear how, or why, only this portion of the larger sample was retained for analysis. It is also unclear how participants were initially recruited for the study and the extent to which participants were sampled randomly from the available population, limiting the external validity of the study. Further, the small sample size makes it difficult to draw reliable conclusions from the data or assess the extent to which age, gender, or other variables (such as practice effects) moderate any reported effects.

Second, some aspects of the research process were not operationalized clearly and consistently. For instance, participants in the original study were required to practice turning the reel on the apparatus “until they had become accustomed to the machine” and were allowed to take “short periods of rest” during the experiment (p. 518). These statements are vague and imprecise. A replication that is sufficiently controlled and that manages the influence of potential confounding variables, such as the amount of practice and standardization of trials, will enhance internal validity.

Third, in terms of data analysis and presentation, data from a subsection of participants is mentioned in passing as exhibiting an overstimulation effect “most strikingly” (p. 523). By modern reporting standards, the presentation of selective elements of a data set in this fine-grained manner would be considered a form of data dredging. However, Triplett’s clear documentation of his experiment and data speak to an attention to detail and care in his work. Equally, the data gathered during the reported trials were not subjected to appropriate statistical interrogation, as those techniques were unavailable at that time in history. Instead, Triplett used his own judgement to describe and tabulate the data. He noted that 20 participants were stimulated positively (i.e., performed better in competition), 10 participants were stimulated adversely (i.e., performed worse in competition), and 10 participants were little affected by competition (i.e., performed roughly equally when alone or in the presence of others).

Presentation and interpretation of data in this way leaves space for bias in interpretation (by both researcher and reader). To scrutinize the original data using modern statistical methods, Strube^33^ reanalyzed Triplett’s original data using both within-subject and between-subject tests. In Triplett’s study, alone and together trials were counterbalanced across two groups (Group A: alone, competition, alone, competition, alone, competition; Group B: alone, alone, competition, alone, competition, alone). For between-subject effects, Strube^33^ compared the counterbalanced alone and competition trials, and while the observed effects were generally in the expected direction, they were not statistically significant. Similarly, within-subject analyses showed little evidence for a competition effect. Only when alone and competition trials were averaged was there a marginally significant effect where participants performed quicker on the competition trials, leading to the conclusion “evidence can be coaxed from the data” (p. 278) with some effort, “indicat[ing] barely a statistical hint of the social facilitation of performance to which his experiment has been credited” (p. 280). Furthermore, while Strube found no moderation by age or gender, the small sample size means this test was underpowered. While the data reanalysis is a welcome and innovative step in revisiting this classic study, unfortunately, these efforts do not overcome other challenges associated with the original study—namely, how participants were recruited, the small (and underpowered) sample size, reporting bias, and standardization of experimental trials. These challenges, we believe, can be overcome with replication of the original study.

### The current research

Replicability is one of the core principles of science^34–36^ and serves as the standard to which scientific claims are held^37^. Replication studies can inform the generalisability of effects. Successful replications provide stronger evidence for the existence of an effect, and lead to more accurate estimates of the effect sizes in meta-analyses. Failed replications can aid in identifying boundary conditions for some effects, or identify false positives in the published literature^38^. Given the historical importance of Triplett’s study, and the recent renewed emphasis on replicability, we argue that there is a strong case for the replication of Triplett’s original demonstration of a social facilitation effect.

We present a single study in which we attempt to replicate Triplett’s original demonstration of the social facilitation effect^2^. Based on Triplett’s diagrams, we constructed a custom-built apparatus, whereby participants turn a crank that causes a marker to travel around a circuit of pulleys. Participants were invited to complete a full circuit (four laps) as fast as they could, both alone and in competition with another participant. The together trials are a competitive coaction setting, drawing on Muller and Butera^25^, and consistent with the wider literature on social facilitation^2,4,39^, and we hypothesized that performance on the *together* trials would be quicker than in the *alone* trials.

We also planned to explore the influence of age and gender, which were not considered in the original study. As previously noted, Strube^33^ did not find gender moderated the results (*p* = 0.087), however, the small sample size in the original study may have meant this test was underpowered. Previous research has shown that females respond less positively to competition, particularly in laboratory/artificial settings for a review see;^39^, so it is important to explore gender as a potential moderator in a well-powered design. Furthermore, we planned to explore the potential of carry-over effects from the together trials onto the subsequent alone trials. Previous work has shown that if participants complete a task in the presence of others, and then complete the task alone, memory effects mean that others might still be “present” phenomenologically during the alone condition^41^, meaning that this condition is not a true alone condition. Therefore, we planned to explore differences between the “true” alone trials (the first trial) and subsequent alone trials that follow a coaction condition.

## Method (as pre-registered)

### Design

The study employed a within-subjects design. The within-subjects factor was trial type with two levels: *alone* vs. *together*. The dependent variable was trial completion speed, as recorded electronically by the purpose-built apparatus.

### Participants

Participants were recruited from schools (*n* = 12) and after-school clubs (*n* = 1) in Ireland. Similar to Triplett^2^, and in line with our Stage 1 pre-registration, only children aged 7–13 years could take part. This analysis reports on usable data from 445 participants (223 boys, 222 girls) who completed the study (*M*_*age*_ = 10.8, *SD* = 1.3).

A priori power analysis using pwr package^42^ in *R* version 4.1^43^, alongside Monte Carlo simulations run on Triplett’s original dataset to confirm the sample size estimation, revealed that, for the main analysis (linear mixed-effects model), in order to detect a small effect size (*f* ^2^ = 0.02) with 80% power a sample of *N* = 400 is required. As such this research had power to detect small effects should they exist.

### Materials

#### Apparatus

Data were collected on a custom-built apparatus as shown in Appendix A. This apparatus consists of two crank and pulley systems side-by-side, such that two participants can engage in the same task concurrently. Turning the crank moves a band of cord along a course (between the pulleys). There is a marker attached to the cord so that participants can visually track the cord as it travels along the course. Performance (dependent variable) is indicated by the time taken for the marker to complete four circuits of the course. Time is recorded by this custom-built apparatus. In the original study^2^, when the crank wheel was turned, this controlled the movement of a stylus which traced a curve on a drum of a kymograph. The direction of this curve corresponded to the rate of turning of the crank providing a high-resolution, graphic representation of changes in speed for each participant for each trial completed. In our apparatus, this information is recorded electronically by sensors attached to an embedded National Instruments myRIO computer^44^.

#### Demographics questionnaire

**Fig. 1:**
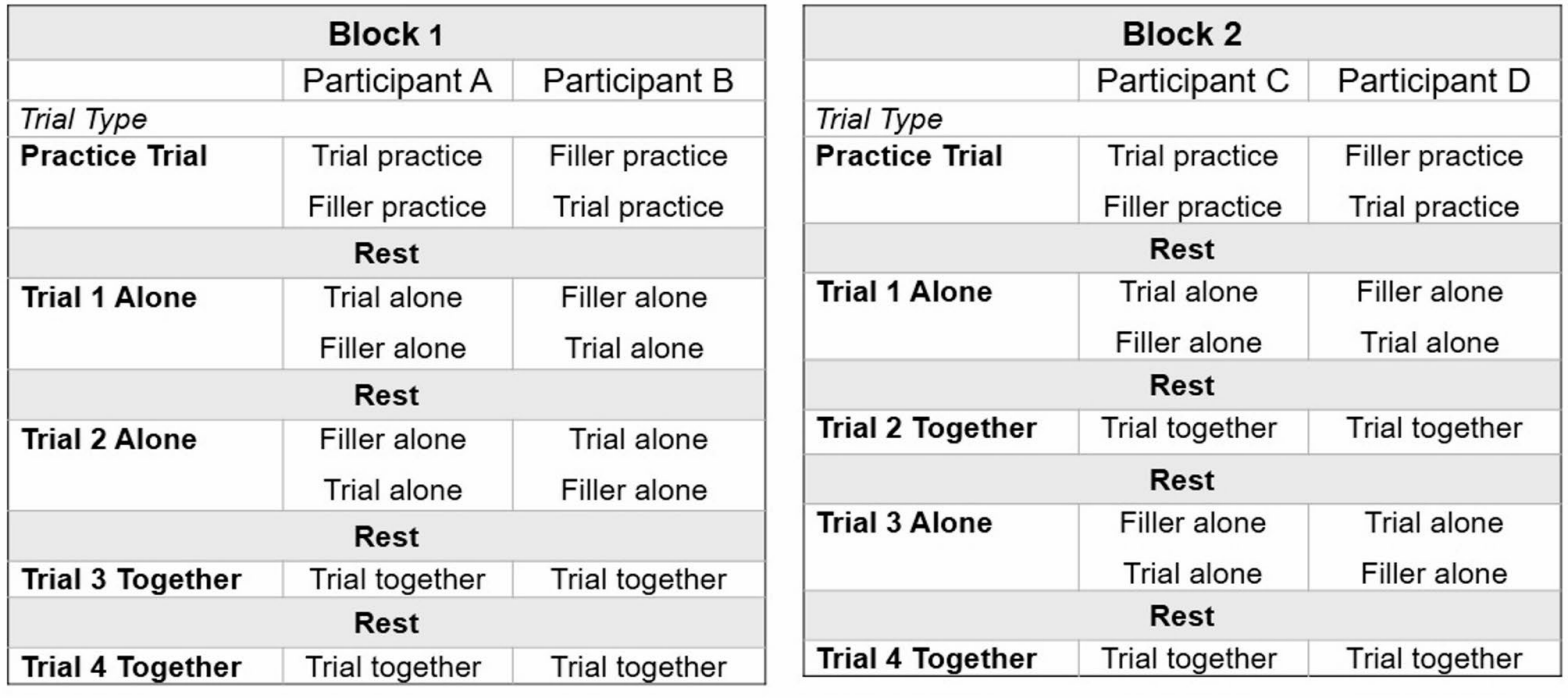
Standardizing task order across participants. Pairs were randomly assigned to each block, and individuals to each sequence (A, B, C, D). Rest periods lasted for 45-seconds.

Basic demographic variables, similar to the original study, (e.g., age, gender) were recorded by the experimenter. Likewise, information relating to trial order (see Fig. 1) and participant handedness (right- or left-handed) were recorded.

#### Procedure

Ethical approval was granted by the institutional ethics board for the planned study procedure (ethics approval number: 2018_12_31_EHS). All data collection took place in educational settings (12 schools, and 1 after-school/homework club). Principals of schools and managers of after-school clubs were emailed information about the study and asked to contact the researchers if interested in taking part. Information sheets and consent forms (including both parental/guardian and participant forms) were provided electronically to schools in advance, and in some cases posted physically through the local mail service. Only children who had parental consent were then invited to take part on the day and were informed orally again about what the study entailed. On the day of participation, children also provided written assent. Participants were reminded that they could withdraw from the study at any stage, even after assent was provided. Participants’ age and gender were recorded. Each participant underwent one practice trial to familiarize themselves with the apparatus, where they turned the wheel to move the flag around the course for one lap. This practice trial was completed alone (with no other participant present) and only one lap allowed to ensure this practice trial was standardized across participants. While one participant completed the practice trial the other practiced the “filler” task out of sight and away from the apparatus. For the filler task participants were asked to balance on one leg for forty-five seconds and the number of times their leg touched the ground was counted. This filler task facilitated the logistics of “alone” and “together” trials for two participants at a time. When one participant was completing the alone task, the other participant was engaged in the filler task allowing them time to rest their arm between competition trials (to reduce possible fatigue) and to keep all children engaged in a standardized task between trials (as opposed to resting quietly or freely moving around).

After both participants had the opportunity to complete one practice trial, there was a forty-five second rest before the first official timed trial began so that participants were fully rested before the first trial. During this rest period, the instructions (i.e., trial instructions and filler task instructions) were reiterated. While it is unclear what children in Triplett’s experiment were told, in the present research participants were told in both the alone and together to “turn the reel as fast as you can to complete four laps. See how quick you can go.” No reference to competition was made, and there were no attempts to create a competitive environment. All participants completed each trial four times, twice alone and twice in a social setting (i.e., in competition with another participant), as specified in Fig. 1. We note that it may not have been possible to fully eliminate the potential for mere presence effects in our alone trials, as participants may have anticipated that their performance would be evaluated and compared against their peers. To mitigate this as much as possible, and to ensure the validity of the alone trial, we set up moveable partitions around three sides of the experiment area (around the two apparatus). During the alone trials, the participant completing the filler task left this area with a research assistant to complete the filler task out of sight of the participant completing the alone trial. Moving to a different area, and the use of movable screens/partitions, ensured that participants could not see each other or each other’s performance during the alone condition/filler task.

To account for possible order effects and/or practice effects, trial order was standardized, resulting in two blocks. Block 1 consisted of the following trials: Alone, Rest, Alone, Rest, Together, Rest, Together. Block 2 consisted of: Alone, Rest, Together, Rest, Alone, Rest, Together. Participant pairs were randomly assigned to either Block 1 or Block 2. Within each block participants were randomly assigned to sequence A or B (Block 1), or C or D (Block 2), which determined who completed the alone trial first and who completed the filler task first. Figure 1 details the sequence for each participant pair. Participants were paired randomly, but participants were required to be of a similar age (within a year) and were matched by gender.

To avoid the potential confound of carryover facilitation effects, the first trial for all participants in both blocks was an Alone trial. Trial 2 was Alone in Block 1 and Together in Block 2, while Trial 3 was Together in Block 1 and Alone in Block 2. The final trial in both blocks was Together. The varied order of trials across blocks was designed to both detect and distinguish between carry-over facilitation effects and practice effects – practice effects would lead to improved performance across trials, whereas facilitation would lead greater improvements in performance for Together trials versus Alone trials. This order also allowed us to test for carry-over effects by comparing response times of Trials 2 and 3 across blocks. If facilitation occured without a carry-over effect, we would observe differences between blocks for both Trials 2 and 3; if facilitation occured with a “carry-over” effect, we would observe differences between blocks for Trial 2 but not for Trial 3.

### Overview of analyses

#### A priori planned analyses

As a preliminary check, we examined if handedness influenced performance. As we anticipated unequal numbers of right-handed vs. left-handed participants we first tested if the variances were equal using Levene’s test for homogeneity of variance. If the variances were equal we planned to proceed with a *t*-test to see if performance differs between people who are right-handed and left-handed (Students *t*-test). If the variances were unequal, we planned to compare the groups using a Welch’s *t*-test. Due to unequal variances Welsh’s *t*-test was conducted. As there was a significant difference in performance between right-handed and left-handed participants, we report the analyses as follows, first, we conduct the analysis in line with our pre-registered data analysis plan without accounting for handedness, next: all proposed analyses were re-conducted with (i) individuals who are left-handed excluded, and (ii) handedness entered as a covariate.

The proposed study employed a within-subjects design. The independent variable was trial type, with two levels (alone versus together), and the dependent variable was performance, operationalized as trial completion speed. To examine our core hypothesis, that the presence of a co-actor led to improved performance on that task (controlling for repeated observations across participants), we conducted a linear mixed-effects model (model 1) with trial type (together, alone) included as a fixed effect, and participant and trial type (together, alone) included as random effects. Participant and trial type were included as pre-registered random effects as our design included pseudoreplications (all participants completed each condition twice so there were multiple measurements in each condition), by including these as random effects in our model it accounted for this non-independence There was no deviation from our Stage 1 analysis plan, and all data and analysis code including sample analysis of simulated data is available on this project’s OSF page at https://osf.io/abf5y/?view_only=1f6f9d74b7094b0d8fc2d4225aa4ea7f.

We anticipated that the possibility of practice effects may have led to improved performance on later trials. To test for this, we conducted a pre-registered second linear mixed-effects model (model 2) that included trial type (together, alone) and trial number (trial 1, trial 2, trial 3, trial 4) as fixed effects, and participant and trial type (together, alone) as random effects. Model 2 additionally tested for a trial type × trial number interaction.

A third pre-registered linear mixed-effects model (model 3) was conducted, which was similar to model 2 but included age and gender as additional predictors to explore if these had effects. To account for the possible differential influence of age and gender, we also included these as interaction terms in our model.

The above models provided an initial test of our hypotheses. To interpret any observed effects, a priori follow-up analyses (e.g., simple slopes) were conducted. Further, it was possible that the competition trials would exert a carry-over effect, such that performance on subsequent alone trials would continue to demonstrate improved performance^21^. To account for this possibility, we standardized the order of trial type across blocks 1 and 2. Specifically, for trial 2 participants in block 1 were alone, and participants in block 2 were together; for trial 3, participants in block 1 were together and participants in block 2 were alone. By isolating trials 2 and 3 we could perform a direct test for carry-over effects. We conducted a 2 × 2 mixed between-within ANOVA to test for an interaction between trial number between groups 1 and 2, and trial type. The between-subjects IV is group (1 vs. 2) and the within-subjects IV is condition (together vs. alone), with follow-up pairwise comparisons with a specific focus comparing the trial 2 alone (block 1) with trial 3 alone (block 2).

Because of differences in presence effects, any order effects may have varied depending on block (1 versus 2). To test this, we conducted a pre-registered 2 × 4 mixed between-within subjects ANOVA with group as the between-subjects IV (group 1 vs. group 2), and trial number as the within subject IV (4 levels, Trial 1- Trial 4), with a full range of post-hoc pairwise comparisons.

#### Post-hoc exploratory analyses

As a small number of participants were paired with a co-actor who had previously competed the task we conducted post-hoc tests to examine if this influenced performance.

Based on our a priori power analysis, our target sample size was *N* = 400. Our final usable sample was *N* = 445. To mitigate the possibility that our study is over-powered and produced false positive results, we conducted a follow-up bootstrapped analysis for each model constraining the resampled sub-samples to *n* = 400.

## Results

### Participants and sample make-up

Data collection ran from April 2023 until October 2024. We recruited a total of *N* = 474 participants (240 girls, 234 boys) in 240 dyads - this included six dyads where one child had already completed the task in order to account for odd numbers. Recruited participants’ ages ranged from 7 to 13 years, *M*_age_ = 10.8, *SD* = 1.3. Duplicate data from these participants were removed.

For our analyses, we omitted data from 29 participants whose data were not usable. Reasons for unusable data include: (a) incomplete data due to technical errors, and (b) external interference/interruptions in the settings where participants completed the study. This left a total usable sample of *N* = 445 (222 girls, 223 boys, *Mage* = 10.8, *SD* = 1.3), and the analyses report on this final sample.

Data collection took place in 13 educational settings in the Southwest of Ireland (12 primary schools and one homework/after-school club). Two schools and the homework club were based in a city (*n*_*total*_ = 140, *n*_*usable*_ = 134), two schools were in medium/small sized towns (*n*_*total*_ = 62, *n*_*usable*_ = 58), two schools were in villages (*n*_*total*_ = 65, *n*_*usable*_ = 64), and there were six remote/rural schools (*n*_*total*_ = 207, *n*_*usable*_ = 189). The overall urban/rural breakdown is *n*_*total*_ = 202 (*n*_*usable*_ = 192) participants from urban areas, and *n*_*total*_ = 272 (*n*_*usable*_ = 253) from rural areas.

We recruited participants from their 4th to 8th year of compulsory primary education (age range 7–13 years, see Table 1), with one small school additionally requesting a student in their 3rd year of education (who met the age requirements) to also take part. The number of participants in each class group are as follows: 1 in their 3rd year of compulsory education (usable = 1), 39 in 4th year (usable = 37), 117 in 5th year (usable = 109), 69 in 6th year (usable = 65), 142 in 7th year (usable = 134), and 106 in 8th year (usable = 99). Full distribution of participants’ ages is displayed in Table 1. There were 46 left-handed (41 with usable data), and 428 right-handed (404 usable) participants in our sample.

**Table 1:** Age distribution of participants in the recruited sample and in the usable (final) sample.

Age	Recruited	Usable/final sample
7	3	3
8	40	36
9	102	94
10	96	91
11	134	129
12	94	87
13	5	5

### Pre-registered analyses

#### Preliminary analyses, effects of age, gender, and handedness

There was no significant difference in age between males (*M* = 10.8, *SD* = 1.24), and females (*M* = 10.8, *SD* = 1.36) in the final sample, *t*(469.8) = −0.73, *p* = 0.466, *d* = 0.07.

There was a significant negative correlation between age and time taken to complete the course, *r* = - 0.34, *p* < 0.001. Older participants were faster at completing the course than younger participants.

We tested if performance on the task varied depending on gender using Welch’s *t*-test (variances were not equal Levene’s test *p* < 0.001). We found significant gender differences in performance *t*(1751.75) = −6.97, *p* < 0.001, *d* = 0.32, with male participants (*M* = 58.6, *SD* = 14.15) taking longer than female participants (*M* = 54.62, *SD* = 10.74) to complete the task.

We then tested if performance on the task varied depending on participant handedness (left vs. right). Levene’s test for homogeneity of variances indicated that variances were not equal (*p* < 0.001), so we tested for handedness differences using Welch’s *t*-test. This revealed a significant difference in time taken to complete the task with left-handed participants taking longer (*M* = 62.1, *SD* = 17.03) than right-handed (*M* = 56.02, *SD* = 12.00) participants, *t*(202.89) = 4.74, *p* < 0.001, *d* = 0.49.

#### Testing the social facilitation effect

In line with our pre-registered analysis plan, we conducted three linear mixed-effects models to test the effect of social facilitation on performance. Model 1 included condition only, model 2 additionally included trial order as a covariate and a condition × order interaction term, while model 3 also included age and gender as covariates. Each model is reported below.

##### Model 1 condition only

We conducted a linear mixed-effects model to test if condition (together vs. alone) influenced performance on the task. Our outcome variable was time taken (in seconds) to complete the course. We included condition (together vs. alone) as a fixed effect. Participant and condition (together, alone; to account for pseudoreplications, multiple measurements in each condition) were included as random effects.

**Fig. 2:**
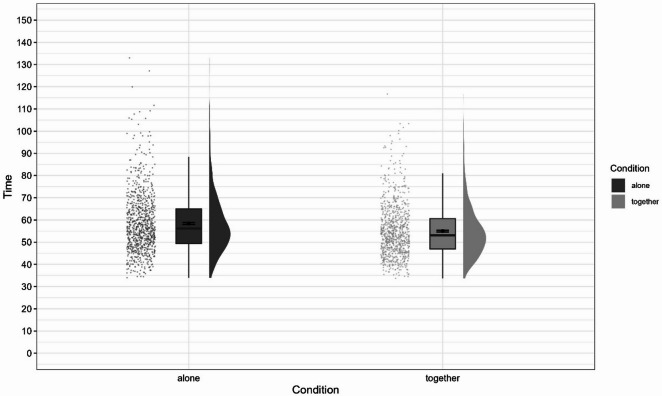
Differences in time taken to complete the course depending on experimental condition.

**Fig. 3:**
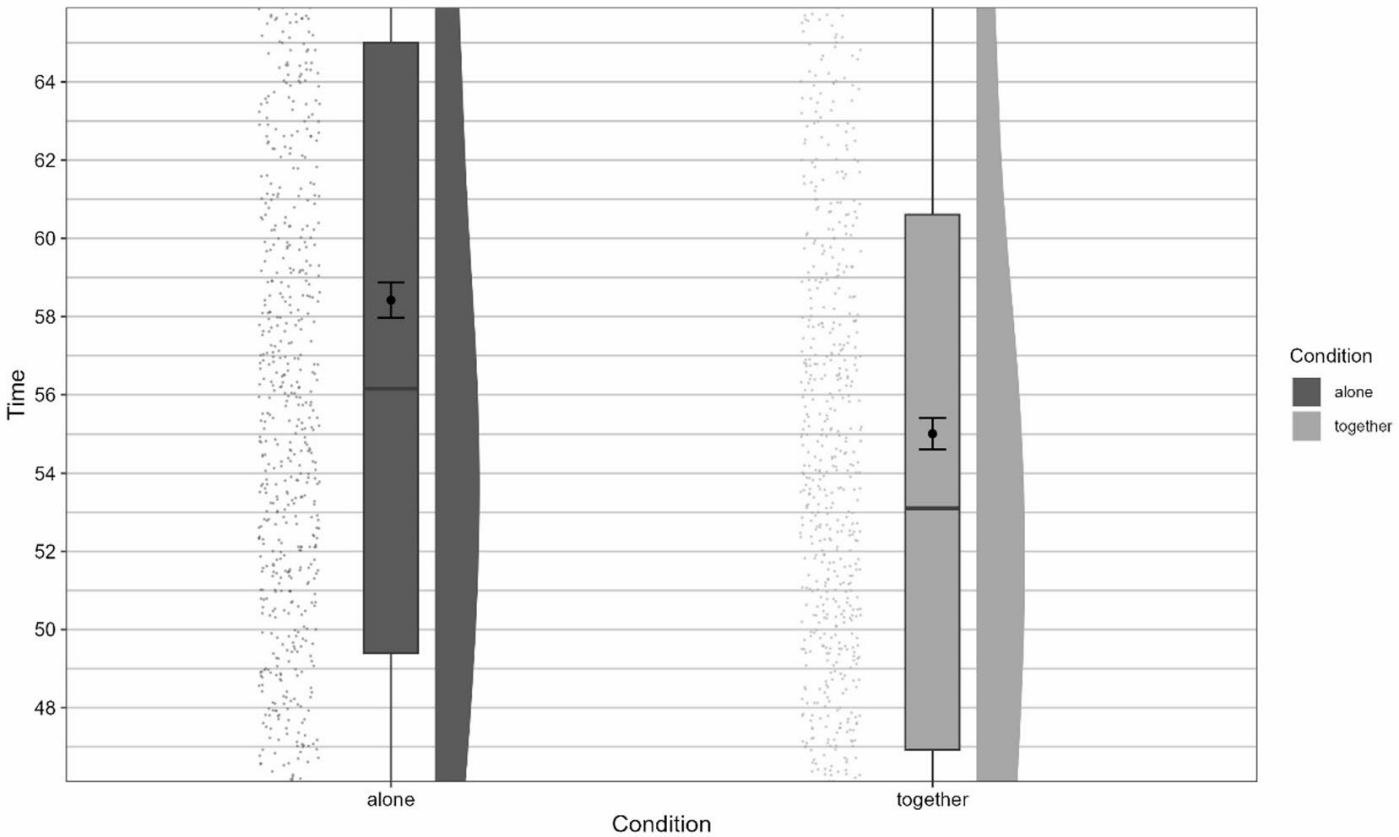
Differences in time taken to complete the course depending on experimental condition (truncated).

Overall, the model significantly predicted time taken to complete the course and provided a better fit to the data than the baseline model, χ^2^(1) = 117.16, *p* < 0.001. Condition (together vs. alone) significantly influenced time, *F*(1, 444) = 133.73, *p* < 0.001, and was a significant predictor in the model, *b* = −3.41, *t*(444) = −11.56, *p* < 0.001. Participants were faster in the together condition than in the alone condition (*M*_alone_ = 58.4, *SD*_alone_ = 13.5, *M*_together_ = 55, *SD*_together_ = 11.8). Figures 2 and 3 show the time taken to complete the course for each condition, Fig. 2 shows the full distribution, while the y-axis in Fig. 3 is truncated to aid interpretation.

##### Model 2 condition with order as interaction term

We conducted a second linear mixed-effects model to test for order effects. The model was the same as the first model but with the inclusion of order as a fixed effect, and the inclusion of an order × condition interaction term (also as a fixed effect).

Overall, the model significantly predicted time taken to complete the course, and provided a better fit to the data, χ^2^(3) = 293.71, *p* < 0.001, (AIC = 11930.6, BIC = 11974.5, Log-likelihood = −5957.3) when compared to the baseline model (AIC = 12218.3, BIC = 12245.8, Log-likelihood = −6104.2), and also compared to the model with condition only, χ^2^(2) = 176.55, *p* < 0.001, (AIC = 12103.2, BIC = 12136.1, Log-likelihood = −6045.6).

Order significantly influenced time, *F*(1, 923.81) = 132.87, *p* < 0.001, and was a significant predictor in the model, *b* = −1.94, *t*(924) = −11.53, *p* < 0.001. Participants were faster on later trials than on earlier trials.

Condition (together vs. alone) significantly influenced time, *F*(1, 1216.84) = 14.92, *p* < 0.001, and was a significant predictor in the model, *b* = −2.67, *t*(1217) = −3.86, *p* < 0.001. When controlling for order and a possible order × condition interaction, participants were faster in the together condition than in the alone condition.

**Fig. 4:**
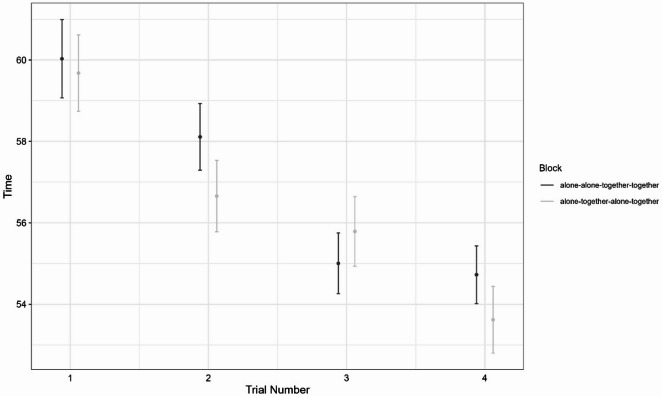
Mean time taken to complete the course for each trial for each experimental block, and each condition; error bars represent standard errors.

There was a significant condition × order interaction, *F*(1, 893.51) = 7.93, *p* = 0.005; and this interaction term was a significant predictor in the model, *b* = 0.68, *t*(894) = 2.82, *p* = 0.005. Simple slopes analyses suggested the effect for order was stronger when participants were alone, *b* = −1.95, *t*(449) = −10.75, *p* < 0.001, than when they were together, *b* = −1.27, *t*(449) = −8.01, *p* < 0.001. This suggests that when participants were alone, practice effects led to a greater improvement in performance compared to when they were together. It may be the case that when participants were together, improvements in performance due to competition/social facilitation effects masked some of the improvement due to practice. Alternatively, this could be an artifact of our experimental set-up. For example, it is possible that the effect of practice diminishes across multiple trials as performance stabilises. In such a case improvements in performance would be greater in earlier trials, compared to later trials. In our study, the first trial for both blocks was an alone trial, and as such, it may not be possible to make an accurate comparison of the effect of practice across condition. Future research employing an alternative block design may be needed to address this. Table 2 shows the means and standard deviations (time taken to complete the course) for each condition for each trial, organised by block. Figure 4 shows the mean time taken to complete the course (and standard error) for each trial and each condition (organized by block). The axes are truncated to aid in interpretation.

**Table 2:** Means and standard deviations for each trial for each block (and each condition).

Trial	Block 1				Block 2			
	Alone		Together		Alone		Together	
	*M*	*SD*	*M*	*SD*	*M*	*SD*	*M*	*SD*
1	60.0	14.5	-	-	59.7	13.8	-	-
2	58.1	12.4	-	-	-	-	56.7	12.6
3	-	-	55.0	11.2	55.8	12.6	-	-
4	-	-	54.7	10.7	-	-	53.6	12.1

##### Model 3 condition, order and covariates

We conducted a third linear mixed-effects model to test for the influence of covariates. The model was similar to model 2 but with age and gender added as covariates, an age × condition interaction term, and a gender × condition interaction term additionally included.

Overall, the model significantly predicted time taken to complete the course, and provided a better fit to the data, χ^2^(7) = 386, *p* < 0.001, (AIC = 11846.3, BIC = 11912.2, Log-likelihood = −5911.2) when compared to the baseline model (AIC = 12218.3, BIC = 12245.8, Log-likelihood = −6103.9), and the condition only model χ^2^(6) = 268.84, *p* < 0.001, (AIC = 12103.2, BIC = 12136.1, Log-likelihood = −6045.6), and provided a better fit than the model with condition, order, and the condition × order interaction term χ^2^(4) = 92.29, *p* < 0.001, (AIC = 11930.6, BIC = 11974.5, Log-likelihood = −5957.3). The full results are displayed in Table 3. As can be seen from Table 3, when controlling for the covariates, and possible interactions, the effect of condition remained: participants in the together condition were faster than participants in the alone condition.

**Table 3:** Coefficients table for model 3. Condition coding: 0 = alone, 1 = together. Gender coding: 0 = male, 1 = female.

	b	SE	df	t	*p*	2.5%	97.5%
(Intercept)	100.91	4.85	445.25	20.80	< 0.001**	110.45	91.37
Condition	−5.76	2.54	500.10	−2.27	0.020*	−0.78	−10.75
Order	−1.94	0.17	924.08	−11.51	< 0.001**	−1.61	−2.27
Age	−3.44	0.44	442.00	−7.78	< 0.001**	−2.57	−4.31
Gender	−4.06	1.15	442.01	−3.54	< 0.001**	−1.80	−6.31
Condition × Order	0.68	0.24	894.60	2.83	< 0.001**	1.15	0.21
Condition × Age	0.34	0.22	442.00	1.52	0.130	0.78	−0.10
Condition × Gender	−1.23	0.58	442.09	−2.11	0.040*	−0.08	−2.37

Interestingly, there was a significant gender × condition interaction. The results of our pre-registered simple slopes analyses suggested the effect of condition was stronger for female participants *b* = −4.07, *t*(665) = −14.22, *p* < 0.001, than for male participants *b* = −2.75, *t*(668) = −7.18, *p* < 0.001.

#### Testing for carry-over effects of presence

In order to test for a carry-over effect from competition trials on subsequent alone trials, we isolated the second and third trials for both blocks, and conducted a pre-registered 2 × 2 mixed between-within factorial ANOVA. The within-subjects IV was condition (together versus alone), and the between-subjects IV was experimental block (block 1 vs. block 2). The order of conditions for trials 2 and 3 for block 1 was alone-together, and the order of conditions for block 2 was together-alone.

There was a main effect for condition *F*(1, 443) = 12.98, *p* < 0.001; and there was no main effect for block *F*(1, 443) = 0.09, *p* = 0.766. There was a significant condition × block interaction effect *F*(1, 443) = 43.31, *p* < 0.001. Tukey’s post-hoc pairwise comparisons revealed that for block 1 (alone first) participants were significantly faster in the together condition than in the alone condition (*p* < 0.001), for block 2 (together first) there was no difference between the alone condition and the together condition (*p* =.161), suggesting a carry-over effect from the together trial (see Table 2; Fig. 4).

#### Testing for order effects depending on block

To test if differences between blocks influenced the presence of order effects, we conducted a pre-registered 4 × 2 mixed between-within-subjects factorial ANOVA, with order as the within-subjects IV (4 levels, trial 1-trial 4), and block as the between-subjects IV (2 levels, block 1 and block 2).

The ANOVA found a main effect for order *F*(3, 1329) = 118.22, *p* < 0.001; no main effect for block *F*(1, 443) = 0.22, *p* =.637, and a significant order × block interaction effect *F*(3, 1329) = 4.7, *p* = 0.003. Tukey’s post-hoc pairwise comparisons revealed that for block 1 trial 1 (alone) was significantly slower than all other trials (all *p-value*s < 0.001), trial 2 (alone) was significantly slower than trials 3 (together) and 4 (together) (*p-value*s < 0.001), while there were no significant differences between trials 3 and 4 (*p* =.999). For block 2, trial 1 (alone) was significantly slower than all other trials (all *p-value*s < 0.001), there was no significant difference between trials 2 (together) and 3 (alone) (*p* =.567), and both were significantly slower than trial 4 (together; *p-value*s < 0.001). Means and standard deviations for each trial for each block are displayed in Table 2.

#### The role of handedness

Initial analysis indicated that there were differences in performance between left-handed and right-handed participants (with right-handed participants taking less time to complete the course than left-handed participants). In anticipation of this, we proposed two alternative sets of analyses in our pre-registered analysis plan. First, we would run the proposed analyses with left-handed individuals excluded. Second, we would include handedness as a covariate in our models. We conducted these analyses and report them below.

##### Right-handed participants only

We ran the same three models as above on our right-handed participants only, model 1: condition only, model 2, condition with order as a covariate and interaction term, and model 3, similar to model 2 but with covariates included.

Overall, model 1 significantly predicted time taken to complete the course, and provided a better fit to the data than the baseline model, χ^2^(1) = 99.07, *p* < 0.001. Condition (together vs. alone) significantly influenced time, *F*(1, 403) = 112, *p* < 0.001; and was a significant predictor in the model, *b* = −3.24, *t*(403) = −10.58, *p* < 0.001, participants were faster in the together condition than in the alone condition (*M*_alone_ = 57.7, *SD*_alone_ = 12.7, *M*_together_ = 54.5, *SD*_together_ = 11.2).

Overall, model 2 significantly predicted time taken to complete the course, and provided a better fit to the data than the baseline model, χ^2^(3) = 259.95, *p* < 0.001, (AIC = 10722.7, BIC = 10765.8, Log-likelihood = −5353.4), when compared to the baseline model (AIC = 10976.7, BIC = 11003.6, Log-likelihood = −5483.3), and also provided a better fit than the model with condition only χ^2^(2) = 160.88, *p* < 0.001, (AIC = 10879.6, BIC = 10911.9, Log-likelihood = −5433.8). Order significantly influenced time, *F*(1, 837.24) = 123.2, *p* < 0.001; and was a significant predictor in the model, *b* = −1.9, *t*(837) = −11.1, *p* < 0.001. Participants were faster on later trials than on earlier trials. Condition (together vs. alone) significantly influenced time, *F*(1, 1113.19) = 13.87, *p* < 0.001; and was a significant predictor in the model, *b* = −2.63, *t*(1113) = −3.72, *p* < 0.001. When controlling for order and a possible order × condition interaction, participants were faster in the together condition than in the alone condition. There was a significant condition × order interaction, *F*(1, 811.29) = 8.11, *p* = 0.005; and this interaction term was a significant predictor in the model, *b* = 0.7, *t*(811) = 2.85, *p* = 0.005. Simple slopes analyses suggested the effect for order was stronger when participants were alone, *b* = −1.95, *t*(449) = −10.75, *p* < 0.001, than when they were together, *b* = −1.27, *t*(449) = −8.01, *p* < 0.001. Again, this suggests that when participants were alone, practice effects led to a greater improvement in performance than when they were together.

Overall, model 3 significantly predicted time taken to complete the course, and provided a better fit to the data, χ^2^(7) = 339.15, *p* < 0.001, (AIC = 10651.5, BIC = 10716.2, Log-likelihood = −5313.8), when compared to the baseline model (AIC = 10976.7, BIC = 11003.6, Log-likelihood = −5483), and the model with condition only χ^2^(6) = 240.08, *p* < 0.001, (AIC = 10879.6, BIC = 10911.9, Log-likelihood = −5433.8), and provided a better fit than the model with condition, order, and the condition × order interaction term χ^2^(4) = 79.2, *p* < 0.001, (AIC = 10722.7, BIC = 10765.8, Log-likelihood = −5353.4). The full results are displayed in Table 4. Interestingly, when controlling for covariates, and possible interactions, the effect of condition was not significant in the right-handed only sample. There was a significant condition × gender interaction. Simple slopes analyses suggests the effect of condition is stronger for female participants *b* = −4.03, *t*(608) = −13.31, *p* < 0.001, than for male participants *b* = −2.45, *t*(602) = −6.31, *p* < 0.001.

**Table 4:** Coefficients table for model 3 when left-handed participants are removed. Condition coding: 0 = alone, 1 = together. Gender Coding: 0 = male, 1 = female.

	B	SE	df	t	*p*	2.5%	97.5%
(Intercept)	96.56	4.92	403.94	19.61	< 0.001**	106.24	86.88
Condition	−4.77	2.67	449.71	−1.78	0.080	0.49	−10.02
Order	−1.90	0.17	837.53	−11.08	< 0.001**	−1.56	−2.24
Age	−3.12	0.45	400.99	−6.99	< 0.001**	−2.24	−3.99
Gender	−3.63	1.14	401.00	−3.17	< 0.001**	−1.38	−5.88
Condition × Order	0.70	0.24	812.24	2.87	< 0.001**	1.18	0.22
Condition × Age	0.26	0.24	400.99	1.12	0.260	0.73	−0.20
Condition × Gender	−1.47	0.60	401.08	−2.43	0.020*	−0.28	−2.66

##### Handedness as a covariate

We ran the same three models as above, but included handedness as a covariate in each as pre-registered, model 1: condition only, model 2, condition with order as a covariate and interaction term, and model 3, similar to model 2 but with covariates included.

Overall, model 1 significantly predicted time taken to complete the course, and provided a better fit to the data than the baseline model, χ^2^(2) = 126.57, *p* < 0.001. Condition (together vs. alone) significantly influenced time, *F*(1, 444) = 133.73, *p* < 0.001; and was a significant predictor in the model, *b* = −3.41, *t*(444) = −11.56, *p* < 0.001, participants were faster in the together condition than in the alone condition (*M*_alone_ = 58.4, *SD*_alone_ = 13.5, *M*_together_ = 55, *SD*_together_ = 11.8). Handedness significantly influenced time, *F*(1, 443) = 9.54, *p* = 0.002; and was a significant predictor in the model, *b* = 5.74, *t*(443) = 3.09, *p* = 0.002.

Overall, model 2 significantly predicted time taken to complete the course, and provided a better fit to the data, χ^2^(4) = 303.09, *p* < 0.001, (AIC = 11923.3, BIC = 11972.6, Log-likelihood = −5952.6) when compared to the baseline model (AIC = 12218.3, BIC = 12245.8, Log-likelihood = −6104.2), and the model with condition only χ^2^(2) = 176.52, *p* < 0.001, (AIC = 12095.8, BIC = 12134.2, Log-likelihood = −6039.4). Order significantly influenced time, *F*(1, 923.83) = 132.88, *p* < 0.001; and was a significant predictor in the model, *b* = −1.94, *t*(924) = −11.53, *p* < 0.001. Participants were faster on later trials than on earlier trials. Condition (together vs. alone) significantly influenced time, *F*(1, 1216.82) = 14.93, *p* < 0.001; and was a significant predictor in the model, *b* = −2.67, *t*(1217) = −3.86, *p* < 0.001. When controlling for order and a possible order × condition interaction, participants were faster in the together condition than in the alone condition. Handedness significantly influenced time, *F*(1, 443) = 9.51, *p* = 0.002; and was a significant predictor in the model, *b* = 5.74, *t*(443) = 3.08, *p* = 0.002. There was a significant condition × order interaction, *F*(1, 893.62) = 7.93, *p* = 0.005; and this interaction term was a significant predictor in the model, *b* = 0.68, *t*(894) = 2.82, *p* = 0.005. Simple slopes analyses suggests the effect for order is stronger when participants are alone, *b* = −1.95, *t*(449) = −10.75, *p* < 0.001, than when they are together *b* = −1.27, *t*(449) = −8.01, *p* < 0.001. As noted above, this may suggest that practice effects lead to a greater improvement in performance when participants are alone than when they are together.

Overall, model 3 (AIC = 11841, BIC = 11912.3, Log-likelihood = −5907.5) significantly predicted time taken to complete the course and provided a better fit to the data when compared to the baseline model, χ^2^(8) = 393.36, *p* < 0.001, (AIC = 12218.3, BIC = 12245.8, Log-likelihood = −6104.2), and to the model with condition only χ^2^(6) = 266.79, *p* < 0.001, (condition only; AIC = 12095.8, BIC = 12134.2, Log-likelihood = −6040.9). Model 3 also provided a better fit than the model with condition, order, and the condition × order interaction term χ^2^(4) = 90.27, *p* < 0.001, (AIC = 11923.3, BIC = 11972.6, Log-likelihood = −5952.6). The full results are displayed in Table 5. Interestingly, when controlling for covariates, and possible interactions, the effect of condition was not significant in the right-handed only sample. Simple slopes analyses suggests the effect of condition is stronger for female participants *b* = −4.07, *t*(665) = −14.22, *p* < 0.001, than for male participants *b* = −2.75, *t*(668) = −7.18, *p* < 0.001.

**Table 5:** Coefficients table for model 3 when handedness is included as a covariate. Condition coding: 0 = alone, 1 = together. Gender Coding: 0 = male, 1 = female.

	*b*	SE	*df*	*t*	*p*	2.5%	97.5%
(Intercept)	99.71	4.82	445.41	20.67	< 0.001**	109.20	90.23
Condition	−5.76	2.54	500.11	−2.27	0.020*	−0.78	−10.75
Order	−1.94	0.17	924.11	−11.51	< 0.001**	−1.61	−2.27
Age	−3.37	0.44	441.00	−7.69	< 0.001**	−2.51	−4.23
Gender	−3.99	1.14	440.37	−3.52	< 0.001**	−1.76	−6.23
Handedness	4.63	1.70	441.00	2.72	0.010*	7.98	1.29
Condition × Order	0.68	0.24	894.69	2.83	< 0.001**	1.15	0.21
Condition × Age	0.34	0.22	442.00	1.52	0.130	0.78	−0.10
Condition × Gender	−1.23	0.58	442.09	−2.11	0.040*	−0.08	−2.37

### Exploratory analyses

#### Testing a potential co-actor effect

We examined, post-hoc, whether or not being paired with a participant who had already completed the task influenced results. We conducted two sets of tests, first we tested if being paired with a participant who had already completed the task influenced performance on the task; second, we tested if being paired with a participant who had already completed the task impacted the effect (if any) of experimental condition. For both tests we found no effect, and concluded that being paired with a participant who has already completed the task does not influence results and as such these participants were retained for the analyses above (for full results of these tests see Appendix B).

### Follow-up bootstrapped analysis

Based on our a priori power analysis our target sample size was *N* = 400. Our final usable sample was *N* = 445. To mitigate the possibility that our study is over-powered and produced false positive results, we conducted a follow-up bootstrapped analysis for each model constraining the resampled sub-samples to *n* = 400. Our bootstrapped analysis resampled 1000 iterations of *n* = 400 from the full usable sample (*N* = 445). The results of the bootstrapped analysis mirrored those reported above. Model 3 provided the best fit (AIC = 10633.2, BIC = 10692.4, Log-likelihood = −5305.6), performing significantly better than model 2, χ^2^(3) = 92.77, *p* < 0.001, (model 2: AIC = 10720, BIC = 10763, Log-likelihood = −5427.4); model 2 performed significantly better than model 1, χ^2^(2) = 150.74, *p* < 0.001, (model 1: AIC = 10866.7, BIC = 10899, Log-likelihood = −5427.4); and model 1 performed significantly better than the baseline model, χ^2^(1) = 113.56, *p* < 0.001 (baseline model: AIC = 10978.3, BIC = 11005.2, Log-likelihood = −5484.1). In the bootstrapped model 1, condition significantly predicted time *b* = −3.36, *t*(399) = −11.06, *p* < 0.001. In the bootstrapped model 2, both order (*b* = −1.91, *t*[829.66] = −10.74, *p* < 0.001), and condition (*b* = −2.58, *t* [1096.49] = −3.54, *p* < 0.001), significantly predicted time, and there was a significant order × condition interaction (*b* = −0.66, *t*[802.99] = −2.61, *p* = 0.010). The results of bootstrapped model 3 are displayed in Table 6.

**Table 6:** Coefficients table for bootstrapped resampled analysis. Condition coding: 0 = alone, 1 = together. Gender Coding: 0 = male, 1 = female.

	*b*	SE	*df*	t	*p*	2.5%	97.5%
(Intercept)	103.29	5.26	399.91	19.63	< 0.001**	113.63	92.94
Condition	−6.33	2.73	446.87	−2.32	0.020*	−0.97	−11.69
Order	−2.00	0.18	830.13	−11.24	< 0.001**	−1.65	−2.36
Age	−3.62	0.48	396.98	−7.50	< 0.001**	−2.67	−4.56
Gender	−4.33	1.23	396.99	−3.52	< 0.001**	−1.91	−6.75
Condition × Order	0.66	0.25	803.72	2.59	0.010*	1.16	0.16
Condition × Age	0.41	0.24	396.98	1.70	0.090	0.89	−0.07
Condition × Gender	−1.15	0.62	397.03	−1.86	0.060	0.06	−2.37

## Discussion

Over 125 years ago Norman Triplett conducted what has often been referred to as the first experiment in social psychology^1^ and showed that completing a task in competition with another participant leads to improved performance compared to completing the task alone. This study was never directly replicated. In response to this, and in line with recent trends in psychological science to examine the replicability of psychological findings, we conducted a single pre-registered study to attempt to replicate Triplett’s original finding, integrating this seminal work with modern techniques (including open science practices and relevant statistical testing). To our knowledge, this is the first pre-registered direct replication of Triplett’s original study^2^. In line with Triplett’s original study^2^, and consistent with the wider literature on social facilitation^3,4^, our findings show that when participants were told to complete the task as fast as possible, participants were quicker in the together condition than in the alone condition. We replicated the original findings presented by Triplett^2^and provide additional evidence for the existence of social facilitation e.g.,^8,9,12^.

This social facilitation effect was relatively robust, that is, even when accounting for other potential influences (including practice effects) we continue to find evidence of this effect. To test for practice effects and potential carry-over effects we varied the order of experimental conditions across two blocks. Specifically, the sequence of conditions for some participants was alone-alone-together-together (block 1), while for other participants it was alone-together-alone-together (block 2). This variation of the sequences allowed us to test and show the presence of a social facilitation effect while statistically controlling for practice effects. In addition, by comparing performance on trials 2 and 3 across blocks we observed an apparent carry-over effect. That is, for trial 2 participants were faster in the together condition than in the alone condition, while for trial 3 performance was similar between the together and alone conditions. This suggests that the improved performance arising from completing the task together “carries over” to the subsequent alone trial^41^. This has implications for research examining this phenomenon and suggests that studies should use a counterbalanced design or otherwise plan for this potential effect.

Based on the original study^2,33^, we expected males to complete the task faster than females. In contrast, within the current sample, females completed the task faster than males on average. Furthermore, the results demonstrated that there was an interaction effect, whereby the social facilitation effect was stronger for females than for males. Acknowledging that no explicit effort was made to construct a competitive environment, this finding was unexpected given that some previous research has suggested that females respond less positively to competition than males^40,45^. However, other research has found no difference between males and females in terms of competitiveness^46^. This underscores the need for further investigation into the role of gender differences in social facilitation and we encourage future research to explore the underlying mechanisms driving this effect. Likewise, the unsurprising finding that older participants were faster than younger participants demonstrates the importance of controlling for age, and for including age-matched pairs, in this type of research.

### Social facilitation or competitive co-action?

While our results were in line with our hypotheses we cannot discern what is driving the observed effects – social facilitation or competition. That is, participants in the together trials may have perceived them as competitive trials – and anecdotally this was often observed by the researchers. Therefore, it is possible that any improvements in performance could be attributed to this perception of competition, rather than mere presence effects.

There is an important theoretical distinction sometimes noted in the social facilitation literature - while some research examining social facilitation includes a co-action task, many definitions of social facilitation focus on the mere presence of another, who is an observer rather than a co-actor^47^. This would suggest that coaction effects (competitive or not) are separate from mere presence effects. Further, while many experimental paradigms including a co-action task aim to examine only the effects of a co-actor, and not the effects of competition, it is difficult to separate the two. Although some experiments ensure participants cannot see each other’s performance^48^, many others do not and instead may inform participants not to compare scores^49^ or compete with one another^50^, or alternatively do not explicitly mention a competitive situation^39^. While the latter examples are typically considered as non-competitive coaction contexts and are considered under the remit of social facilitation effects for systematic reviews see^4,51^, there is still potential for participants to compare their performance and compete. Indeed, this may be the case with Triplett’s original experiment. Despite giving rise to the social facilitation literature, we do not know what instructions participants were given, and considering Triplett’s interest in competition then perhaps this experiment tests competitive co-action effects, not necessarily social facilitation effects (as defined by the mere presence literature).

There are a range of perspectives on competitive co-action effects. According to self-awareness theory^8,52,53^, competing in the presence of others leads to increased self-awareness, and any differences between one’s own performance and the performance of competitor(s) are highly salient. This theory proposes that individuals are motivated to perform in line with perceived standards – where a standard is mental representation of what is perceived to be a “correct” behavior^52,53^. In situations of competitive co-action, the perceived standard is influenced by the performance of the competitor(s). Where there is a discrepancy between the performance of the self, compared to the perceived standard, this results in an aversive state^52,53^. Individuals are motivated to improve performance in order to avoid this aversive self-other discrepancy^8^.

Where self-awareness theory emphasizes the self in relation to perceived standards (where these standards can be influenced by the presence of competitors), social comparison theory focuses on direct comparison with others^26,54,55^. Festinger argued that people are driven to improve performance to minimize discrepancies between themselves and a target. This leads to competitiveness and competitive behavior aimed at protecting a sense of superiority^26,54^. A range of factors that can impact competitiveness have been identified, including: (i) whether comparisons are upward (a more skilled target) or downward (a less skilled target); (ii) the relevance of the activity to the individual; (iii) the degree of similarity between the individual and target; (iv) the closeness to the target; (v) incentive structures; and (vi) proximity to a standard, to name a few^54^. In presenting their social comparison model of competition, Garcia et al.^54^, integrate these diverse influences, distinguishing between individual factors, such as (i) to (iv), and situational factors, such as (v) and (vi).

### Limitations and future directions

It is important to note that the observed findings do not definitively support any specific theoretical explanation of the effect e.g.,^12,18–25^. For instance, we do not know if the observed effects were due to an increase in generalized drive^14^, expected evaluation^19^, or changes in challenge vs. threat appraisals for an overview see;^56^. From our study design it is also not possible to discern whether our results are due to social facilitation/co-action effects or due to *competitive* coaction e.g.,^8,25^. More research is needed to delineate the cause of the phenomenon and understand this complex social-psychological process more fully. Despite this, our findings contribute to the empirical evidence for the phenomenon and provide additional insights into the role of other variables, particularly the importance of counterbalancing for carry-over effects, and the influence of gender and age.

Further limitations of our study stem from the constraints of our design. Specifically, we employed a within-subjects design, where all participants completed each condition twice. While completing each condition twice allowed us to test for order/practice effects, it also introduced a potential source of error that may have influenced performance in the alone trials. Participants would have been aware that both they, and their partners, would complete the alone trials again. It is possible that this knowledge led participants to anticipate their performance would be evaluated and compared against their peers, leading to potential mere presence effects. While we cannot rule out this possibility, the presence of both the social facilitation/competitive coaction effects, and the carry-over effects described above do suggest that our alone conditions were meaningfully different from our together conditions and that any effect of anticipated evaluation on the alone trials is likely minimal. Future research should employ different designs to fully examine the social facilitation/competitive coaction effect without this potential confound. Another limitation is that in our two blocks there was an alone trial first and a together trial last. While we were able to demonstrate that order/practice effects and social facilitation/competitive coaction effects were both present, additional trial sequences would be able to further differentiate between these two effects.

The current study has several methodological strengths. First, our study benefits from increased statistical power by analyzing data from over four-hundred participants — compared to forty participants reported in Triplett’s^2^ analysis. With this larger sample, our study was generally sufficiently powered to detect a small effect — though we note that including more predictors in our model may have led to reduced statistical power^35^, perhaps contributing to some of the mixed findings observed (i.e., no significant effect for condition in model 3 when left-handed participants are removed). Second, our methods were pre-registered and standardized. We standardized the practice trials, the rest periods, the filler task, and the instructions provided to participants. We also employed clear and transparent a priori inclusion criteria and recruitment strategy. Third, our operationalization and measurement of the variable of interest involved a sensor that directly recorded the rotations of the crank and the time taken to complete the course, providing a highly accurate (and high resolution) measure of participants’ performance on the task. By employing a larger sample, pre-registration, and precise measurement techniques, our study provides a strong foundation for future research while also highlighting important considerations for study design and analysis.

## Conclusion

Our findings replicate the original findings presented by Triplett^2^ and provide further evidence for social facilitation and/or competitive coaction effects^2,4,8,12^. The methods, materials, and approach used in this research provide a foundation for future work that can test some of the key theoretical debates in the literature, including if it is competitive co-action, co-action, or mere presence that is driving the effects. In replicating this classic finding we showed that, despite recent concerns regarding the quality and replicability of classic social psychological findings, this classic effect does appear to be a “real” and robust effect.

## Data Availability

All data files are available at the OSF page for this project at https://osf.io/abf5y/?view_only=1f6f9d74b7094b0d8fc2d4225aa4ea7f.

## Code availability

All analysis code is available at the OSF page for this project at https://osf.io/abf5y/?view_only=1f6f9d74b7094b0d8fc2d4225aa4ea7f.

## Laboratory log

The Laboratory Log detailing the data collection periods (April 2023 to October 2024) is available at the OSF page for this project at https://osf.io/yxhwu?view_only=1f6f9d74b7094b0d8fc2d4225aa4ea7f.

## References

[1] Amabile, T. M., Goldfarb, P. & Brackfleld, S. C. Social influences on creativity: Evaluation, coaction, and surveillance. *Creat Res. J.* **3**, 6–21 (1990).

[2] Triplett, N. The dynamogenic factors in pacemaking and competition. *Am. J. Psychol.* **9**, 507–533 (1898).

[3] Aronson, E., Wilson, T. D. & Akert, R. M. *Social Psychology* (Pearson Education International, 2005).

[4] Bond, C. F. & Titus, L. J. Social facilitation: A meta-analysis of 241 studies. *Psychol. Bull.* **94**, 265–292 (1983). 6356198

[5] Weinberg, R. S. & Gould, D. *Foundations of Sport and Exercise Psychology* (Human Kinetics, 2019).

[6] Haines, H. & Vaughan, G. M. Was 1898 a great date in the history of experimental social psychology? *J. Hist. Behav. Sci.* **15**, 323–332 (1979). 11608235 10.1002/1520-6696(197910)15:4<323::aid-jhbs2300150405>3.0.co;2-i

[7] Karau, S. J. & Williams, K. D. in *In Soc. Psychol. Revisiting Class. Stud*. 11–26 (eds Smith, J. R. & Haslam, S. A.) (SAGE, 2012).

[8] Rhea, M. R., Landers, D. M., Alvar, B. A. & Arent, S. M. The effects of competition and the presence of an audience on weight lifting performance. *J. Strength. Cond Res.* **17**, 303–306 (2003). 12741867 10.1519/1533-4287(2003)017<0303:teocat>2.0.co;2

[9] Allport, F. H. The influence of the group upon association and thought. *J. Exp. Psychol.* **3**, 159–182 (1920).

[10] Dashiell, J. F. An experimental analysis of some group effects. *J. Abnorm. Soc. Psychol.* **25**, 190–199 (1930).

[11] Seitchik, A. E., Brown, A. J. & Harkins, S. G. in *Oxf. Handb. Soc. Influ.* 183–203Oxford University Press, (2017).

[12] Zajonc, R. B. Social facilitation. *Science* **149**, 269–274 (1965). 14300526 10.1126/science.149.3681.269

[13] Tolman, C. W. The varieties of social stimulation in the feeding behaviour of domestic chicks. *Behaviour* **30**, 275–286 (1968). 5677597 10.1163/156853968x00351

[14] Zajonc, R. B., Heingartner, A. & Herman, E. M. Social enhancement and impairment of performance in the cockroach. *J. Pers. Soc. Psychol.* **13**, 83–92 (1969).

[15] Halfmann, E., Bredehöft, J. & Häusser, J. A. Replicating roaches: A preregistered direct replication of Zajonc, Heingartner, and herman’s (1969) social-facilitation study. *Psychol. Sci.* **31**, 332–337 (2020). 32017670 10.1177/0956797620902101

[16] Hull, C. L. *Principles of Behavior* (Appleton-Century Crofts, 1943).

[17] Spence, K. W. *Behavior theory and conditioning*. vii, 267Yale University Press, (1956). 10.1037/10029-000

[18] Cottrell, N. B. in *In Exp. Soc. Psychol*. (eds McClintock, C. G.) (Holt, Rinehart & Winston, 1972).

[19] Cottrell, N. B., Wack, D. L., Sekerak, G. J. & Rittle, R. H. Social facilitation of dominant responses by the presence of an audience and the Mere presence of others. *J. Pers. Soc. Psychol.* **9**, 245–250 (1968). 5666972 10.1037/h0025902

[20] Weiss, R. F. & Miller, F. G. The drive theory of social facilitation. *Psychol. Rev.* **78**, 44–57 (1971).

[21] Baron, R. S. in *Adv. Exp. Soc. Psychol.* (ed. Berkowitz, L.) 19, 1–40Academic Press, (1986).

[22] Belletier, C., Normand, A. & Huguet, P. Social-facilitation-and-impairment effects: from motivation to cognition and the social brain. *Curr. Dir. Psychol. Sci.* **28**, 260–265 (2019).

[23] Huguet, P., Galvaing, M. P., Monteil, J. M. & Dumas, F. Social presence effects in the Stroop task: further evidence for an attentional view of social facilitation. *J. Pers. Soc. Psychol.* **77**, 1011–1025 (1999). 10573878 10.1037//0022-3514.77.5.1011

[24] Sanders, G. S., Baron, R. S. & Moore, D. L. Distraction and social comparison as mediators of social facilitation effects. *J. Exp. Soc. Psychol.* **14**, 291–303 (1978).

[25] Muller, D. & Butera, F. The focusing effect of self-evaluation threat in Coaction and social comparison. *J. Pers. Soc. Psychol.* **93**, 194–211 (2007). 17645395 10.1037/0022-3514.93.2.194

[26] Festinger, L. A theory of social comparison processes. *Hum. Relat.* **7**, 117–140 (1954).

[27] de Castro, J. M. Family and friends produce greater social facilitation of food intake than other companions. *Physiol. Behav.* **56**, 445–455 (1994). 7972393 10.1016/0031-9384(94)90286-0

[28] Izuma, K., Saito, D. N. & Sadato, N. Processing of the incentive for social approval in the ventral striatum during charitable donation. *J. Cogn. Neurosci.* **22**, 621–631 (2010). 19320552 10.1162/jocn.2009.21228

[29] Sommer, R., Wynes, M. & Brinkley, G. Social facilitation effects in shopping behavior. *Environ. Behav.* **24**, 285–297 (1992).

[30] Anderson-Hanley, C., Arciero, P. & Snyder Social facilitation in virtual reality-enhanced exercise: competitiveness moderates exercise effort of older adults. *Clin. Interv Aging*. **275**10.2147/CIA.S25337 (2011). 10.2147/CIA.S25337 PMC3212419 22087067

[31] Rosenbloom, T., Shahar, A., Perlman, A., Estreich, D. & Kirzner, E. Success on a practical driver’s license test with and without the presence of another testee. *Accid. Anal. Prev.* **39**, 1296–1301 (2007). 17920854 10.1016/j.aap.2007.03.015

[32] Feldman, R. S. *Social Psychology* (Prentice Hall, 1995).

[33] Strube, M. J. What did Triplett really find? A contemporary analysis of the first experiment in social psychology. *Am. J. Psychol.* **118**, 271–286 (2005). 15989124

[34] Hempel, C. G. & Oppenheim, P. Studies in the logic of explanation. *Philos. Sci.* **15**, 135–175 (1948).

[35] Meehl, P. E. Appraising and amending theories: the strategy of Lakatosian defense and two principles that warrant it. *Psychol. Inq.* **1**, 108–141 (1990).

[36] Open Science Collaboration. Estimating the reproducibility of psychological science. *Science* **349**, aac4716 (2015). 26315443 10.1126/science.aac4716

[37] Nosek, B. A., Spies, J. R. & Motyl, M. Scientific utopia: ii. restructuring incentives and practices to promote truth over publishability. *Perspect. Psychol. Sci.* **7**, 615–631 (2012). 26168121 10.1177/1745691612459058 PMC10540222

[38] Nosek, B. A. & Lakens, D. Registered reports: a method to increase the credibility of published results. *Soc. Psychol.* **45**, 137–141 (2014).

[39] MacCracken, M. J. & Stadulis, R. E. Social facilitation of young children’s dynamic balance performance. *J. Sport Exerc. Psychol.* **7**, 150–165 (1985).

[40] Niederle, M. & Vesterlund, L. Gender and competition. *Annu. Rev. Econ.* **3**, 601–630 (2011).

[41] Markus, H. The effect of Mere presence on social facilitation: an unobtrusive test. *J. Exp. Soc. Psychol.* **14**, 389–397 (1978).

[42] Champely, S. et al. pwr: Basic functions for power analysis. (2017).

[43] R Core Team. R: A language and environment for statistical computing. at: (2021). https://www.R-project.org/

[44] Doering, E. & NI myRIO Project Essentials Guide. (2016). at: https://education.ni.com/teach/resources/92/ni-myrio-project-essentials-guide

[45] Gneezy, U. & Rustichini, A. Gender and competition at a young age. *Am. Econ. Rev.* **94**, 377–381 (2004).

[46] Dreber, A., von Essen, E. & Ranehill, E. Outrunning the gender gap—boys and girls compete equally. *Exp. Econ.* **14**, 567–582 (2011).

[47] Uziel, L. Individual differences in the social facilitation effect: A review and meta-analysis. *J. Res. Personal*. **41**, 579–601 (2007).

[48] Hall, E. G. & Bunker, L. K. Locus of control as a mediator of social facilitation effects during motor skill learning. *J. Sport Exerc. Psychol.* **1**, 332–335 (1979).

[49] Bird, A. M. Effects of social facilitation upon females’ performance of two psychomotor tasks. *Res. Q. Am. Assoc. Health Phys. Educ. Recreat*. **44**, 322–330 (1973).

[50] Martens, R., Landers, D. M. & and Coaction effects on a muscular endurance task. *Res. Q. Am. Assoc. Health Phys. Educ. Recreat*. **40**, 733–737 (1969). 5262102

[51] van Meurs, E., Greve & Strauss, B. Jonaand Moving in the presence of others – a systematic review and meta-analysis on social facilitation. *Int. Rev. Sport Exerc. Psychol.* 17, 980–1012 (2024).

[52] Silvia, P. & Duval, T. Objective self-awareness theory: recent progress and enduring problems. *Personal Soc. Psychol. Rev. - SOC. PSYCHOL. REV.* **5**, 230–241 (2001).

[53] Duval, S. & Wicklund, R. A. *A theory of objective self awareness*. x, 238Academic Press, (1972).

[54] Garcia, S. M., Tor, A. & Schiff, T. M. The psychology of competition: a social comparison perspective. *Perspect. Psychol. Sci.* **8**, 634–650 (2013). 26173228 10.1177/1745691613504114

[55] Crusius, J., Corcoran, K. & Mussweiler, T. in *In Theor. Soc. Psychol. Second Ed*. 165–187 (eds Chadee, D.) (Wiley, 2022). 10.1002/9781394266616.ch7

[56] Blascovich, J., Mendes, W. B., Hunter, S. B. & Salomon, K. Social ‘facilitation’ as challenge and threat. *J. Pers. Soc. Psychol.* **77**, 68–77 (1999). 10434409 10.1037//0022-3514.77.1.68

